# Assessing chronic stress in wild mammals using claw-derived cortisol: a validation using European badgers (*Meles meles*)

**DOI:** 10.1093/conphys/coad024

**Published:** 2023-05-10

**Authors:** H Bobby Fokidis, Taylor Brock, Chris Newman, David W Macdonald, Christina D Buesching

**Affiliations:** Department of Biology, Rollins College, 1000 Holt Avenue, Winter Park, Florida, 32789-4499, USA; Department of Biology, Rollins College, 1000 Holt Avenue, Winter Park, Florida, 32789-4499, USA; Wildlife Conservation Research Unit, Department of Zoology, University of Oxford, The Recanati-Kaplan Centre, Tubney House, Abindgon Rd, Tubney, OX13 5QL, UK; Wildlife Conservation Research Unit, Department of Zoology, University of Oxford, The Recanati-Kaplan Centre, Tubney House, Abindgon Rd, Tubney, OX13 5QL, UK; Irving K. Barber Faculty of Science, University of British Columbia, Okanagan campus, 3187 University Way, Kelowna, British Columbia, V1V1V7, Canada

**Keywords:** stress, steroids, glucocorticoids

## Abstract

Measuring stress experienced by wild mammals is increasingly important in the context of human-induced rapid environmental change and initiatives to mitigate human-wildlife conflicts. Glucocorticoids (GC), such as cortisol, mediate responses by promoting physiological adjustments during environmental perturbations. Measuring cortisol is a popular technique; however, this often reveals only recent short-term stress such as that incurred by restraining the animal to sample blood, corrupting the veracity of this approach. Here we present a protocol using claw cortisol, compared with hair cortisol, as a long-term stress bio-indicator, which circumvents this constraint, where claw tissue archives the individual’s GC concentration over preceding weeks. We then correlate our findings against detailed knowledge of European badger life history stressors. Based on a solid-phase extraction method, we assessed how claw cortisol concentrations related to season and badger sex, age and body-condition using a combination of generalized linear mixed models (GLMM) (*n* = 668 samples from 273 unique individuals) followed by finer scale mixed models for repeated measures (MMRM) (*n* = 152 re-captured individuals). Claw and hair cortisol assays achieved high accuracy, precision and repeatability, with similar sensitivity. The top GLMM model for claw cortisol included age, sex, season and the sex*season interaction. Overall, claw cortisol levels were significantly higher among males than females, but strongly influenced by season, where females had higher levels than males in autumn. The top fine scale MMRM model included sex, age and body condition, with claw cortisol significantly higher in males, older and thinner individuals. Hair cortisol was more variable than claw; nevertheless, there was a positive correlation after removing 34 outliers. We discuss strong support for these stress-related claw cortisol patterns from previous studies of badger biology. Given the potential of this technique, we conclude that it has broad application in conservation biology.

## Teaser

We established a measure of cortisol, a hormone involved in stress, in claws from wild mammals. Whereas blood samples yield short-term information compromised by trapping stress, this technique permits measurements during preceding weeks. We used data from a long-term European badger study to assess findings, concluding this approach has wide application.

## Introduction

Wild animals experience environmental variability and stochasticity, which can drive adaptation through optimizing body condition, physiology and behaviour to best endure current conditions, which gives individuals important fitness benefits (Noonan *et al.,* 2018). Such plasticity is a response to a broad range of environmental, social and demographic factors that influence an individual over its lifetime, accounting for the non-genetic component of individual variation (Forsman, 2015; Bright Ross *et al.,* 2021). Glucocorticoids (GCs), such as cortisol, mediate plasticity by promoting physiological adjustments during environmental perturbations (Guindre-Parker, 2018). Secreted by the adrenal glands, GCs regulate energy availability through glucose and protein metabolism (Brillon *et al.,* 1995) and serve to align an animal’s physiological state with environmental conditions. During particularly adverse circumstances, GCs are secreted at concentrations above baseline; however, despite being critical for maintaining homeostasis during acute challenges, long-term (i.e. chronic) GC elevation has deleterious health effects, that can lower fitness (Schoenle *et al.,* 2018). Many species therefore modulate GC release to avoid over-secretion when conservation of energy is paramount, for example when breeding or during hibernation (Wingfield *et al.,* 1998; Vella *et al.,* 2020). Social interactions add greater complexity to the GC profile with reproductive behaviours, antagonistic interactions and social hierarchies all having been associated with changes in GC secretion (Raulo and Dantzer, 2018). Finally, there can be intrinsic differences in GC levels based on demographics such as sex and age (Crespi *et al.,* 2013). Consequently, an individual’s GC titer is an amalgam of many factors, and interpretation is made yet more challenging by the difficulty of discerning acute from chronic GC levels (Sheriff *et al.,* 2011).

Methods for measuring steroids include both invasive blood sampling and non or less- invasive methods such as, feces, urine and saliva sampling. However, all of these represent concentrations at short timescales (minutes to hours) that can be impacted by many concomitant factors like recent capture or evasion of a predator (Millspaugh and Washburn, 2004; Fokidis, 2016). These methods return only a single time-point for interpreting a long-term GC pattern. In contrast, recently keratinized tissues (e.g. hair and claws) have been used as a means to assess GC secretion over longer timescales (e.g. weeks to months) (Macbeth *et al.,* 2010; Mack and Fokidis, 2017). Here, plasma GCs are implicitly deposited in growing claw and hair tissues where they are thought to become embedded during the keratinization process (Terwissen *et al.,* 2013; Burnard *et al.,* 2016; Ralph and Tilbrook, 2016; Shi *et al.,* 2021). Consequently, these measures may represent the average hormone titer over the period of tissue growth independent of any recent fluctuations. However, more research is needed to preclude the contribution of locally synthesized cortisol, which occurs in hair follicle (Ito *et al.,* 2005) and can overestimate levels (Sergiel *et al.,* 2020). Whether this occurs in the claw matrix is currently unknown. The measurement of hormone in hair is further complicated by potential seasonal variation in pelage growth rates and hair lengths (Maurel *et al.,* 1986), whereas claw growth remains relatively consistent in most species examined (Ethier *et al.,* 2010). Although used in turtles (Baxter-Gilbert *et al.,* 2014); chameleons (Matas *et al.,* 2016); cattle (Comin *et al.,* 2014); seals (Karpovich *et al.,* 2020; Crain *et al.,* 2021); cats (Contreras *et al.,* 2021); and dogs (Mack and Fokidis, 2017; Jasmine *et al.,* 2018), little research has investigated how claw GC concentrations differ within and among free-living individuals, nor explored the sources of this variation.

Here we used European badgers (*Meles meles;* hereafter ‘badger’) as a model species, to test whether claw cortisol might reflect known stressors in free-ranging animals. Badgers are medium-sized generalist carnivores in the family Mustelidae. Across Europe, badger social systems vary from near solitary to group-living, with group size driven by availability of regional food resources being exploited, principally earthworm (*Lumbricus terrestris*) availability (Johnson *et al.,* 2002). Where groups form, they occupy large shared burrow complexes called setts (Noonan *et al.,* 2015a; Tsunoda *et al.,* 2018). Badger physiology and reproductive biology exhibit strict seasonal patterns. In late summer, badger metabolism becomes anabolic but switches back to catabolic in late winter (McClune *et al.,* 2015). Highly altricial cubs are born around mid-February, but only emerge from their burrows after reaching 6 weeks of age, when they interact increasingly with other group members (Fell *et al.,* 2006), and are fully weaned by mid- to end of May. The main mating season coincides with a post-partum oestrus, with blastocycsts undergoing delayed implantation for ca 10 months (Sugianto *et al.,* 2021), with superfecundation and superfoetation possible (Yamaguchi *et al.,* 2006), which may result in multiple paternity of litters (Annavi *et al.,* 2014b). Thus, claw cortisol levels may vary between the sexes and during different stages within this complex reproductive cycle.

Furthermore, cortisol has a well-known and complex relationship with the immune system which can potentially inform about susceptibility to disease. In the UK, badgers are recognized as a reservoir for bovine tuberculosis (bTB) which places them in direct conflict with the cattle industry, leading to government-sanctioned culling interventions (Godfray *et al.,* 2013). Thus cortisol may inform how culling-related stresses can exacerbate bTB prevalence by suppressing the immune system (Carter *et al.,* 2007). Badger responses to natural sources of stress have also been investigated extensively: for example, weather conditions affect their survival (Macdonald *et al.,* 2010; Nouvellet *et al.,* 2013), activity regimes (Noonan *et al.,* 2015b), body condition (Bright Ross *et al.,* 2021), pace of life (Bright Ross *et al.,* 2020) and senescence patterns (Sugianto *et al.,* 2020; van Lieshout *et al.,* 2021a). Furthermore, their promiscuous and polygynandrous mating system combined with extra-group paternity (Annavi *et al.,* 2014a) and endocrine mechanisms to assure mating success (Sugianto *et al.,* 2021) can add to their allostatic load; which can be compounded by mounting an immune reactions to bTB (Bilham *et al.,* 2017) or other physiological challenges (Sin *et al.,* 2014; Tsai *et al.*, 2021, Bilham *et al.,* 2018). Cortisol in badgers can vary with these kinds of environmental and disease factors (George *et al.,* 2014; Agnew *et al.,* 2016).

Attempts to measure stress responses in badgers using cortisol levels in serum, faecal and hair samples have previously shown seasonal variation and sex differences (Schutz *et al.,* 2006; Barja *et al.,* 2012; George *et al.,* 2014; Agnew *et al.,* 2016). However, trapping stress and baiting (Sun *et al.,* 2015) can impact interpretations of instantaneous serum and faecal cortisol, respectively (George *et al.,* 2014). Therefore, establishing a biomarker that can transcend capture stress is desirable.

Since 1987, a continuous trapping program at a high-density badger population at Wytham Woods in Oxfordshire, UK has documented the life histories and sociality of over 1800 individuals from birth to death over four seasonal trapping sessions per year (Macdonald and Newman, 2002; Macdonald *et al.,* 2009, 2015). Using this study system, we were able to develop and validate the use of claw and hair cortisol assays to determine how concentrations vary on population- and individual-levels in response to i) demographic parameters; ii) body condition; iii) reproductive status; iv) socio-spatial factors; v) temporal factors; and vi) capture history. We also investigate how claw cortisol levels relate to those obtained using the longer-established hair cortisol approach. We then use our results to elucidate whether claw cortisol suits exploration of how intrinsic and extrinsic factors may influence cortisol patterns in this species, and the potential to generalize the utility of this technique to other mammals.

## Methods

### Badger trapping and sampling

Claw samples were collected during routine badger trapping sessions in Wytham Woods (51°46′26″ N, 1°19′19″ W) between Sept 2017 and Nov 2019. Badgers were captured (for details see Sun *et al.,* 2015) in late spring/early summer (late May—early June, hereafter spring; i.e. ca. 2 months after the end of the primary mating season, but coinciding with cubs becoming independent), late summer/early autumn (early September, hereafter summer; after ca 2 months of seasonal scarcity of food due to the dry summer, but coinciding with the end of the catabolic metabolic period), and late autumn/early winter (mid to late November, hereafter autumn; i.e. ca 4 weeks after the onset of reproductive quiescence (Sugianto *et al.,* 2018) and the anabolic metabolism period. The number and demographic parameters of sampled badgers are shown in Table 1. Traps were placed and baited with peanuts in known sett locations with the goal of capturing as many individuals as possible (Noonan *et al.,* 2015b). Traps were checked early in the morning each day (before 8 am) and captured animals transported to a field station for sedation with 0.2 ml ketamine hydrochloride/kg body weight by intramuscular (quadriceps) injection, for further details see (McLaren *et al.,* 2005; Thornton *et al.,* 2005; Sun *et al.,* 2015; Sugianto *et al.,* 2019a). Later the same afternoon, processed badgers were released at the site of capture. As badger social groups often utilize more than one sett and badgers in this study population frequently visit neighboring groups, usually without animosity (Ellwood *et al.,* 2017), individuals were assigned residency at the sett where they were caught most often (see Sugianto *et al.*, 2019b) and sett was included as a spatial identifier in the models. Population size was estimated following an enhanced Minimum Number Alive procedure, incorporating inter-census trapping efficiency variation (Bright Ross *et al.,* 2022). Thereafter, individuals were assigned to social groups following rules given in (Annavi *et al.,* 2014b) as modified in (Sugianto *et al.,* 2019b), to provide social group sizes.

**Table 1:** Sample sizes for European badgers (*M. meles*) across trapping seasons and demographic groups. Numbers represent total capture events (number of unique badgers)

		Males		Females	
Year	Season	Cubs	Adults	Cubs	Adults
2017	Summer	2 (2)	26 (25)	3 (3)	20 (20)
	Autumn	1 (1)	16 (16)	2 (2)	17 (17)
					
2018	Spring	12 (12)	41 (32)	14 (13)	30 (26)
	Summer	8 (8)	48 (47)	14 (12)	59 (56)
	Autumn	6 (6)	36 (36)	9 (9)	23 (22)
					
2019	Spring	17 (17)	36 (36)	10 (10)	48 (44)
	Summer	14 (14)	28 (27)	13 (12)	48 (47)
	Autumn	8 (8)	24 (23)	6 (5)	29 (28)
					
Total		68 (68)	255 (242)	71 (66)	274 (260)

Age was derived in one of two ways: Age was known absolutely for badgers first caught and tattooed (hereafter ID) as cubs (72.3% in this study). For individuals first caught and tattooed as adults, age was estimated with tooth wear (Harris *et al.,* 1992; Bright Ross *et al.,* 2020). Sex was established by examining external genitalia, which were further characterized to provide a reproductive status. These reproductive categories are dry or moist vulva for females, and very ascended, ascended, intermediate, descended and very descended testes for males (Sugianto *et al.,* 2018). In females, recent lactation was determined using the length and diameter of teats (Dugdale *et al.,* 2011), and was categorized as either not lactated, lactated or lactating. Badgers were weighed, and the total body length measured to enable the calculation of a morphometric body condition index (BCI). Additionally, a subcutaneous fat score with categories from 1 (emaciated) to 5 (very fat) was assessed by dorsal palpation which provides a more direct metric of fat storage (Bright Ross *et al.,* 2021). Finally, the number of times an animal was captured within the timeframe of this specific study was also recorded.

For each individual, the middle three digits on the right front paw were clipped below the quick (i.e. onychostroma). Paired claw samples from individual badgers were sampled from both left and right forepaws (*n* = 14) or from black and yellowish-coloured claws (*n* = 19) to investigate whether claw location or melanin influenced cortisol concentrations. In some cases, additional samples were collected from the same three digits on the left front paw, and from the remaining two digits on each front paw. Because badgers wear down the claws on their back feet naturally almost to the quick when digging, no samples could be collected. Samples from the same individual were pooled to provide a sufficient volume for cortisol extraction. Samples of dorso-lateral body hair, including follicles was also collected and in some cases, additional samples were collected from the ventro-lateral, mid-dorsal and femoral regions. Badgers typically replace their pelage once a year from July to December and there is no seasonal variation in hair density, unlike other fur-bearing species (Maurel *et al.,* 1986). Both claw and hair samples were dry stored until further processing. All handling and sampling procedures were approved by the Animal Welfare and Ethical Review Board (AWERB) of Oxford University’s Zoology Department and conducted under the Animals (Scientific Procedures) Act, 1986 (PPL: 30/3379) and Natural England licenses (2018–34 017-SCI-SCI).

### Sample extraction

Both claw samples (3–4 mm length from tip) and hair samples (cut into 1–4 mm tip fragments that excluded any follicles) were first cleaned of surface contamination by sonication in deionized (DI) water for 15 min, and then in 85% ethanol for two minutes after which were dried at room temperature overnight. Claw samples were then ground to a powder using a handheld attrition mill (Glas-Col LLC, Atlanta, GA, USA) to improve endogenous cortisol recovery (Mack and Fokidis, 2017). Both sample types were then stored at 20°C until extraction. All claw and hair samples were weighed, and 50 mg were extracted by suspension in 88% HPLC-grade methanol (MeOH) in a volume to sample mass ratio of 19:1 with 1 mm zirconium oxide beads, and then homogenized in a bead homogenizer (settings: 12 m/s for 30 s, Omni Bead Ruptor 24, Omni International, Kennesaw, GA, USA) and left overnight at 4°C. Samples were then centrifuged at 3000 g for 10 min at 4°C and the supernatant collected and diluted with 10 mL of deionized water. Cortisol was extracted using a solid phase extraction (SPE) method with unendcapped carbon-bonded silica C18 filter column cartridges (Agilent Technologies, Santa Clara, CA, USA) on a vacuum manifold at a constant flow rate of c. 2 drops per second. Columns were first primed with 3 mL of 100% ethanol (EtOH), then equilibrated with 10-mL deionized water before loading the diluted 10-mL sample. Next, 10 mL of 40% MeOH was used to remove fats (e.g. triglycerides, cholesterols and fatty acids) from the sample that could interfere with the assay. Columns were then run dry, and cortisol was eluted using 5 mL of 90% MeOH. Samples were dried in a speed vacuum concentrator (ThermoFisher Scientific Inc, Pittsburgh, PA, USA) at 50°C for 6 h. All SPE extractions included a solvent blank as a negative control. Dried extracts were stored at -20°C until assayed together within 27 days.

### Cortisol analysis

Cortisol concentration was quantified using an enzyme-linked immunosorbent assay (ELISA) kit (Arbor Assays Inc, Ann Arbor, MI, USA). The manufacturers reported assay specifications are sensitivity = 17.3 pg/mL, limit of detection = 45.4 pg/mL, intra-assay precision = 8.8%, inter-assay precision = 8.1%, cross-reactivities, dexamethasone = 18.8%, prednisolone = 7.8%, corticosterone = 1.2%, cortisone = 1.2% and all other tested steroids including androgens, progestins and estrogens < 0.1%. Dried extracts were reconstituted using 2 μL of absolute EtOH and 60 μL of the kit assay buffer. The assay was performed as per manufacturer’s instructions. Final concentrations were calculated using raw absorbance data interpolated from the standard curve using GraphPad Prism version 6.0 (GraphPad Software Inc, La Jolla, CA, USA). All samples and standards were run in duplicate, with any claw and hair samples from the same individual run on the same 96-well plate. Each plate (n = 35 plates) contained the same pooled samples, with all pools derived from subsamples from multiple individuals, to determine assay precision and repeatability, here presented as percentages for inter-assay (i.e. between plates) and intra-assay (i.e. within plates) coefficients of variation (% CV), respectively. Sensitivity (i.e. minimal detection limit) was defined as a difference of two standard deviations between the means of the blank standard and the pooled claw samples.

### Assay validations

We conducted four assay validations for use on badger claw and hair. 1) To determine whether other components within the claw or hair matrix biased cortisol measurement, differences between the slopes of the assay standard curve and serially diluted samples (linear range: 1:1 to 1:128 with assay buffer) were tested using linear regression of log-transformed concentrations with *a priori* differences in slopes less than 10% being considered acceptable. Differences between slopes of the lines were tested using likelihood ratio tests of the slope variances. 2) We tested whether increased sampling loading (range: 1.7 to 100.2 mg) increased endogenous cortisol recovery using logistic regression analysis. 3) Recovery was tested by measuring exogenous cortisol (“spikes”) added to pooled extracts at a range of concentrations (from 1.2 to 126 n/ml) and assessed using Pearson’s correlation between the cortisol added and recovered. 4) A subset of pooled claw and hair extracts were pretreated with dextran-coated charcoal (DCC; 3:1 sample by mass) overnight to eliminate endogenous cortisol as a negative control.

### Statistical analyses

Claw cortisol data were analysed using mixed models conducted both with and without fitting random effects. The BCI was calculated using standardized residuals from an ordinary least square’s regression of body mass by total body length for all captures (Bright Ross *et al.,* 2021). Prior to analysis, all continuous data were first tested for adherence to both normality and homoscedasticity assumptions (i.e. equal variance), and log transformed if necessary (Supplementary Table S1). Outlier data points were identified using Chauvenet’s criterion, and analyses were run both with and without outliers present. However, as removing outliers (n = 6) did not alter the results for any model, only the complete data are presented. Two methods were used to ensure no multicollinearity between predictor variables: Pearson correlations and variance inflation factors (VIFs) between each pair of continuous variables, with r > 0.7 and VIF < 3.0 indicating collinearity.

As a preliminary step, we began with a *coarse* analysis using generalized linear mixed models (GLMM) to evaluate relationships between the log-transformed response variable (claw cortisol) and the full range of predictor explanatory variables as fixed effects across the total number of claw samples collected (*n* = 668 samples from 273 unique individuals), also applying relevant interactions determined *a priori* (Supplementary Table S1). We then conducted a *fine-scale* analysis using mixed models for repeated measures (MMRM) with badger ID as a random effect to investigate changes in log-transformed claw cortisol values (response variable) within individuals that had been captured at least twice (*n* = 152). Individual animals were trapped at varying times, and this introduces missing data (e.g. some animals were caught twice whereas others were trapped 10 times) and the MRMM method averts the biases associated with unequal or missing data (Detry and Ma, 2016). Both analyses also included a global model containing all predictor variables (Supplementary Table S1) as main effects.

We then built models starting with the demographic variables (sex and age) and adding attributes in subsequent models (i.e. forward selection) while including potentially biologically relevant interactions. To select the most parsimonious model, Akaike’s information criteria (AIC) were used to rank all models, including a null model (i.e. intercept only), where the best-fit model has the lowest AIC score that relates the most variance explained by the fewest number of model parameters. The ΔAIC score for each model was compared to the best-fit model with scores > 2 having low support. The AIC weight of evidence for each model (ω_i_) was also presented as a relative measure of model fit to these observed data.

Badgers often have either black or yellowish-coloured claws and thus paired *t*-tests were used to determine if the presence of melanin influences cortisol concentrations. Similarly, paired *t*-tests were used to determine differences between samples collected from the left and right forepaws. To assess the relationship between hair and claw cortisol concentrations, Pearson’s correlations were performed on untransformed raw data for that subset of individuals for which both hair and claw collected concurrently (*n* = 431). Furthermore, one-way analysis of variance (ANOVA) was used to compare cortisol concentrations from hair obtained from different body areas, with Tukey’s post-hoc tests comparing regions. For all statistical tests, the alpha level for significance was *p* < 0.05 and analyses were conducted using SPSS (SPSS Inc, San Jose, CA, USA).

## Results

636 individual badgers were captured providing 668 claw and 631 hair samples in total (including recaptures) and with sex ratio close to parity for both adults and cubs (Table 1). A regression of body mass on total body length [mass = −8.46 + 0.0236*length] was robust (goodness of fit: *R^2^* = 0.852; *F _1667_* = 3844.32; *p* < 0.001) and provided standardized residuals for a BCI.

**Figure 1:**
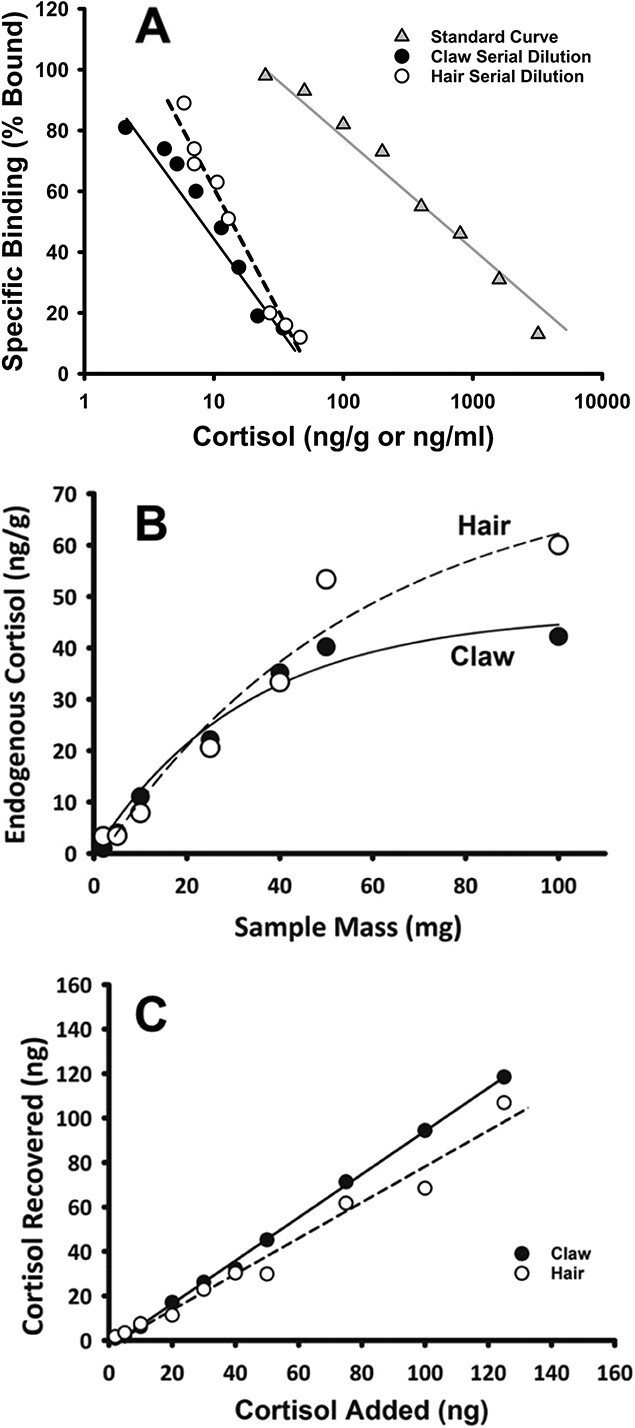
Comparisons of cortisol assays for claw and hair from European badgers. (A) Serial dilution of claw (y = 33.24–0.421log(x)), but not hair samples (y = 43.205–0.529log(x)) were not significantly different from the cortisol assay standard curve (y = 2810.662–0.453log(x)). (B) Cortisol amount recovered from both pooled claw and nail samples increased curvilinearly with mass of the sample. (C) Increased recovery of known (“spiked”) cortisol concentrations in claw (y = 0.935x + 0.71) compared to hair (y = 1.351 + 0.43) samples.

### Cortisol assays

Both the claw and hair cortisol ELISA assays achieved high accuracy (*r* = 0.921 and 0.873, respectively; *n* = 9 each; both *P* ≤ 0.001), precision (13.1% and 16.3% CV, respectively) and repeatability (7.4% and 9.5% CV, respectively). Sensitivity of the claw and hair ELISA assays was similar at 32 and 33 pg/mL, respectively. A serial dilution of pooled claw samples exhibited a linear pattern (*F* = 7.35, *P* = 0.004) with a slope that differed from the cortisol assay standard curve by 7.4%, which is below the 10% *a priori* threshold and not statistically significant (*F _1,6_* = 1.34; *P* = 0.29). (Fig 1A). Serial-diluted pooled hair samples were also linear (*F* = 3.19, *P* = 0.41), but the differences in slope (15.5%) were significantly different from the standard curve (*F _1,6_* = 53.41; *P* ≤ 0.001; Fig 1A). The difference in slope between the hair and claw samples was 22.7%, which was significant (*F _1,6_* = 112.038, *p* < 0.001) and well beyond the 10% threshold (Fig 1A). Increased sample loading (i.e. higher sample mass) resulted in a logistic increase in cortisol measured from both pooled claw and hair samples with the curvilinear range up to 50 mg (Fig. 1B). Hair cortisol concentrations per unit mass were typically higher than in claw, however the latter fit the curvilinear pattern more predictably (claw *R^2^* = 0.859 vs. hair *R^2^* = 0.788; both n = 7 and *p <* 0.018; Fig. 1B). There was also increased average recovery of known (“spiked”) cortisol concentrations in claw extracts compared to hair extracts (claw 84.7% *vs.* hair 79.0%), but recovery in both was correlated to the amount of cortisol that was added to the sample pools (claw r = 0.896, vs. hair r = 0.852; both *n* = 10 and *P* ≤ 0.001; Fig. 1C). Pretreatment with DCC effectively removed endogenous cortisol in both claw and hair sample pools (0.02–0.06 ng/g) to a concentration comparable to the solvent blank (both t ≤ 0.407, *p* ≥ 0.562).

### Model selection

**Figure 2:**
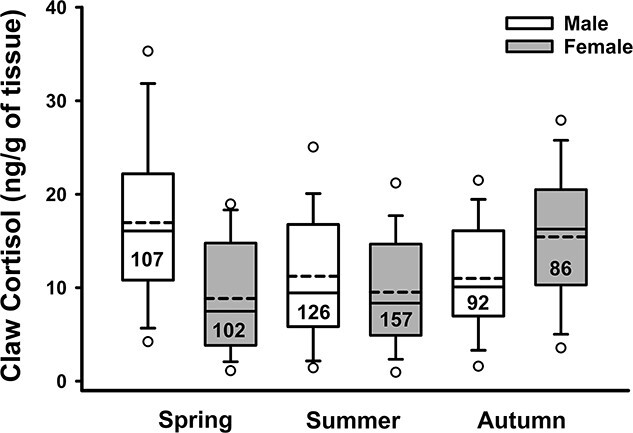
Box plots for the coarse analysis indicating claw cortisol concentrations in male and female European badgers (*M. meles*) sampled during two-week trapping efforts in Wytham Woods, England between Sept 2017 and Nov 2019. Seasons correspond to Spring (late May—early June); Summer (early September) and Autumn (mid to late November). The top and bottom of each box represents the 25% and 75% quartiles (Q1 and Q3), with the solid and dashed inside line representing the median and mean, respectively. The bottom and top error bars indicate the 5% and 95% range and the points represent the 1% to 99% range of values. Numbers within the box indicate sample sizes.

The coarse analysis tested between 15 separate models, including the global and null, with six of these being supported based on our ΔAIC criteria of ≤ 2.00 (Table 2). The top model included badger age, sex, season and the sex*season interaction as predictor variables (Table 2). Overall, claw cortisol concentration was significantly higher among males than among females (Table 3); however, this was strongly influenced by season, particularly in the spring sampling periods (Fig. 2). In contrast, females had higher levels than males in autumn, with no sex-differences apparent in the summer (Fig. 2). Despite a trend for older animals, especially females, to have higher levels, age was not a significant predictor of cortisol concentrations (Table 3). There was also support for models containing either social group size (ΔAIC = 1.66), year (ΔAIC = 1.70) and BCI (ΔAIC = 2.00) as parameters (Table 2). Year, BCI and social group size did not influence claw cortisol concentrations significantly (both β ≤ 0.001; *p* ≥ 0.715). A global model that included all variables lacked support (ΔAIC = 39.50), as did a null model that included only the intercept (ΔAIC = 72.38). Similarly, inclusion of additional variables associated with attributes such as reproductive status, social group, or experience of being captured (Supplementary Table S1) did not improve model support (Table 2).

**Table 2:** Generalized linear mixed model selection results for the coarse analysis (*n* = 668) of factors affecting claw cortisol concentrations in European badgers (*M. meles*)

Model	Candidate models	*k*	AIC	ΔAIC	*ω*	*R^2^*
**1**	Sex + Age + Season + (Sex^*^Season)	**7**	**105.57**	**0.00**	**0.32**	**0.37**
**2**	Sex + Age	**3**	**107.19**	**1.62**	**0.14**	**0.35**
**3**	Sex + Age + (Sex^*^Age)	**6**	**107.21**	**1.64**	**0.14**	**0.34**
**4**	Sex + Age + GroupSize + (Sex^*^GroupSize)	**7**	**107.23**	**1.66**	**0.13**	**0.30**
**5**	Sex + Age + Season + Year	**5**	**107.27**	**1.70**	**0.13**	**0.29**
**6**	Sex + Age + Season + BCI	**5**	**107.56**	**2.00**	**0.12**	**0.29**
7	Sex + Age + BCI + Fat + Genitalia + Lactation	7	109.15	3.58	0.05	-
8	Sex + Age + BCI + Fat	5	109.15	3.58	0.05	-
9	Sex + Age + Season + BCI + (Sex^*^Season^*^ BCI)	10	110.05	4.49	0.03	-
10	Season + GroupSize	3	110.82	5.25	0.02	-
11	Sex + Age + Season + Year + (Sex^*^Season^*^Year)	10	111.64	6.08	0.02	-
12	Sex + Age + BCI + Fat + Genitalia + Lactation + Sett + GroupSize	9	118.07	12.50	0.00	-
13	Global Model (all variables)	12	145.07	39.50	0.00	-
14	Sex + Age + BCI + Fat + Genitalia + Lactation + Sett + GroupSize + Season + Year	11	158.31	57.74	0.00	-
15	Null Model (intercept only)	1	177.95	72.38	0.00	-

Presented are the Akaike information criteria (AIC); the number of model parameters (k); the AIC difference between model and the most supported model (ΔAIC); the Akaike weight of evidence supporting each model (w), and the adjusted coefficient of determination for models where ΔAIC ≤ 2.00 (R2). Bold numbers denote supported models where the ΔAIC ≤ 2.00.

**Table 3:** Parameter estimates for the top generalized linear mixed model from the coarse analysis predicting claw cortisol in European badgers

Parameter	β	*SE*	*P*
Intercept	1.08	0.18	**< 0.001**
Sex	−0.435	0.22	**0.013**
Age	0.09695	0.02	0.055
Season	−0.102	0.05	**0.024**

Shown here are parameter coefficients (β) and their standard error (SE). Bold numbers denote significant parameters where *P* ≤ 0.05.

**Figure 3:**
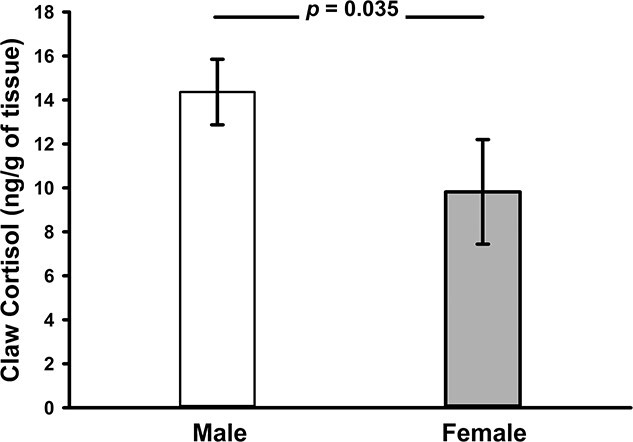
Bar plot indicates significant differences in claw cortisol concentrations between male and female European Badgers (*M. meles*) sampled from Wytham Woods, England as part of the fine-scale analysis (see Methods for details).

**Figure 4:**
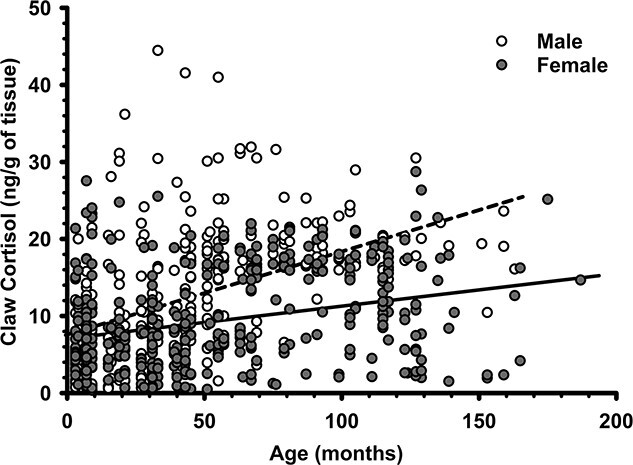
Scatterplot for the fine-scale analysis indicating claw cortisol concentrations in European badgers (*M. meles*) of varying ages. Age was based on mark-recapture data and/or estimation based on tooth wear (see Methods).

**Figure 5:**
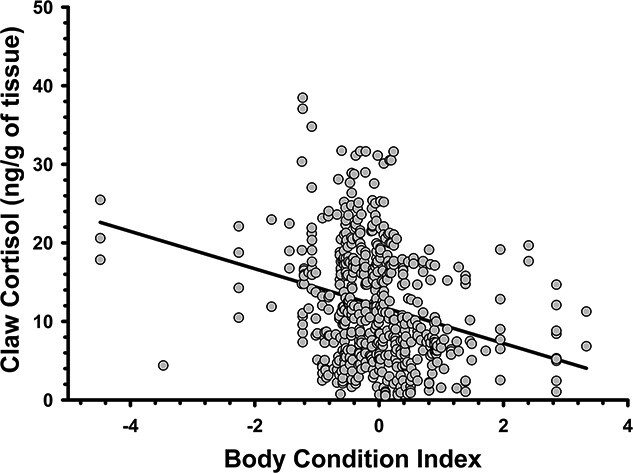
Relationship between claw cortisol concentrations and body condition index (BCI) scores in European badgers (*M. meles*). BCI is derived from standardized residuals of a least squares’ regression of body mass onto total body length (see Methods).

**Figure 6:**
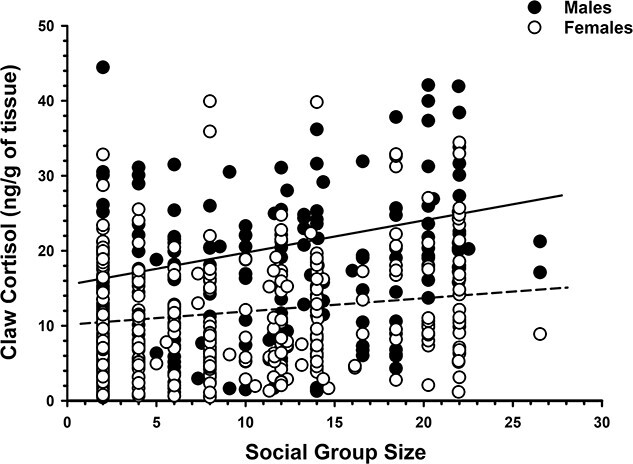
Relationship between claw cortisol concentrations and social group size in both male and female European badgers (*M. meles*). Size of badger social groups was estimated using a novel minimum number alive estimate (see Methods). Claw cortisol concentrations are positively correlated with group sizes in males (solid line: *r* = 0.30, *P* = 0.046), but not females (dashed line: *r* = 0.12, *P* = 0.366).

Of the 15 models tested in the fine-scale analysis, only three had significant support (Table 4). As in the coarse analysis, the top model included both sex and age but also BCI and fat score as predictors (Table 5). Claw cortisol concentrations were significantly higher in males than in females (Fig. 3; Table 5), and in older versus younger badgers (Fig. 4; Table 5). Individuals with a higher BCI (Fig. 5) had lower claw cortisol concentrations, but this effect did not carry over to fat score (Table 5). The fine-scale analysis also supported two other models: one that excluded fat score and instead contained season and the three-way interaction of sex^*^season^*^BCI; and another containing sex, age and social group size, along with the interaction of sex^*^group-size (Table 4). There was a significant positive effect of social group size on claw cortisol concentrations (β = 0.162; *p* = 0.040), but this was significantly pronounced in males, but not females (Fig. 6). However, other parameters (fat, season) did not influence claw cortisol concentrations significantly (all β ≤ 0.001; *p* ≥ 0.281). There was again a lack of support for both the global model (ΔAIC = 49.23) and the null model (ΔAIC = 209.21) and including additional attributes (Supplementary Table S1) did not improve model standing (Table 4).

**Table 4:** Mixed model for repeated measures selection results for the fine-scale analysis (*n* = 152) of factors affecting claw cortisol concentrations in European badgers (*M. meles*) from Wytham Woods, England

Model	Candidate Models	*k*	AIC	ΔAIC	*ω_i_*	*R^2^*
1	ID + Sex + Age + BCI + Fat	**6**	**174.21**	**0.00**	**0.65**	**0.39**
2	ID + Sex + Age + Season + BCI + (Sex^*^Season^*^ BCI)	**11**	**175.59**	**1.38**	**0.32**	**0.36**
3	ID + Sex + Age + GroupSize + (Sex^*^GroupSize)	**8**	**176.18**	**1.97**	**0.11**	**0.31**
4	ID + Sex + Age + BCI + Fat + Trapped	8	180.38	6.17	0.03	-
5	ID + Sex + Age + Season + (Sex*Season)	8	203.97	29.75	0.00	-
6	ID + Sex + Age + BCI + Fat + Genitalia + Lactation + Sett + GroupSize	10	211.37	37.16	0.00	-
7	ID + Sex + Age + Season + BCI	6	213.78	39.57	0.00	-
8	Global Model (all variables)	13	223.44	49.23	0.00	-
9	ID + Season + GroupSize	4	244.01	69.80	0.00	-
10	ID + Sex + Age + Season + Year	6	256.22	82.01	0.00	-
11	ID + Sex + Age + (Sex^*^Age)	7	263.64	89.43	0.00	-
12	ID + Sex + Age + BCI + Fat + Genitalia + Lactation + Sett + GroupSize + Season + Year	12	300.19	125.98	0.00	-
13	ID + Sex + Age	4	307.93	133.72	0.00	-
14	ID + Sex + Age + Season + Year + (Sex^*^Season^*^Year)	11	359.95	185.73	0.00	-
15	Null Model (intercept only)	1	383.43	209.21	0.00	-

Presented are the AIC; the number of model parameters (k); the AIC difference between model and the most supported model (ΔAIC); the Akaike weight of evidence supporting each model (w), and the adjusted coefficient of determination for models where ΔAIC ≤ 2.00 (R2). Bold numbers denote supported models where the ΔAIC ≤ 2.00.

**Table 5:** Parameter estimates for the top mixed model for repeated measures from the fine-scale analysis predicting claw cortisol in European badgers

Parameter	β	*SE*	*P*
Intercept	1.472	0.29	**< 0.001**
ID	0.022	0.15	0.403
Sex	−0.177	0.17	**0.035**
Age	0.239	0.15	**0.008**
BCI	−0.462	0.67	**0.029**
Fat score	−0.034	0.48	0.203

Shown here are parameter coefficients (β) and their standard error (SE). Bold numbers denote significant parameters where *P* ≤ 0.05.

### Cortisol variation in claw and hair samples

**Figure 7:**
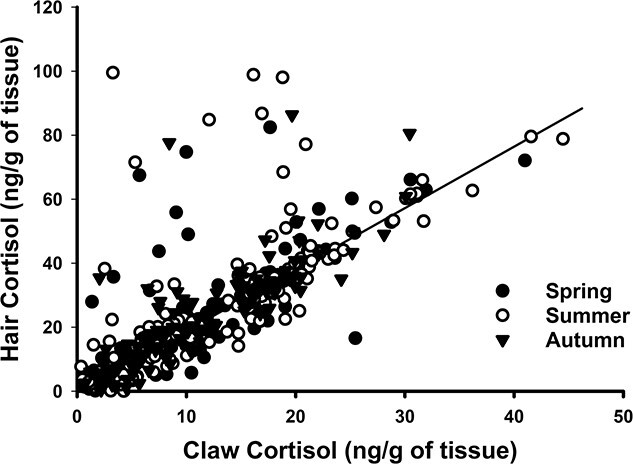
Pearson’s correlation of cortisol concentrations extracted from both claw and hair samples from European badgers (*M. meles*). Symbols indicate samples collected in different seasons. Trendline is significant at r = 0.772 with n = 431 samples and p < 0.001.

Within individual badgers, there was no differences between black and yellow claws (paired *t* = 0.746; *n* = 19; *P* = 0.375). Similarly claw cortisol levels derived from the left and right forepaw were not significantly different (paired *t* = −1.013; *n* = 14; *P* = 0.314). The site of hair collection did not influence hair cortisol concentrations (*F _1,17_* = 1.004; *P* = 0.183). Claw and hair cortisol concentrations were significantly positively correlated (*r* = 0.772, *P* ≤ 0.001, *n* = 431; Fig. 7). Hair cortisol was more variable than claw cortisol with 34 separate outliers compared to the 17 outliers for claw cortisol. Nevertheless, a positive correlation was still observed even after removing all hair and claw outliers (*r* = 0.521, *P* = 0.007, *n* = 382).

## Discussion

Sampling cortisol, or other steroids, from claws provides many practical advantages to field biologists. These include their easy collection that is minimally invasive for the animal (although likely to require physical restraint); simple storage under normal field conditions; and no immediate need for further processing, centrifugation, or freezing. The unique utility of analyzing hormones stored in claws is the ability to infer hormone secretion patterns over longer periods compared to blood, saliva, or feces. The growth of claws in mammals is associated with rapid division of keratinocytes in the terminal matrix of the dermis that lines the ungual processes of the phalanx (Ethier *et al.,* 2010), and steroids that passively diffuse through this vascularized region and become embedded once the unguis becomes cornified into a claw sheath (Homberger *et al.,* 2009; Mack and Fokidis, 2017). Nail growth rates vary across species but estimates from American badgers (*Taxidea taxus*) suggest a rate of 0.26–0.28 mm/day with total claw replacement after 130–140 days (Ethier *et al.,* 2010). Claw samples in this study taken from the distal portion of the claw ranged in length from 16 to 19 mm equating to about 62–68 days of growth using the reported rates for *T. taxus* (Ethier *et al.,* 2010). Although badger taxonomy is not completely resolved and includes polyphyly (Marmi *et al.,* 2004; Kinoshita *et al.,* 2017; Koepfli *et al.,* 2018), the conservative growth rates described here are comparable to other carnivores (Ethier *et al.,* 2010). Thus, claw concentrations may inform about average cortisol levels spanning approximately 2–2.5 months prior to capture.

All claw samples tested had measurable amounts of cortisol but were consistently lower than amounts reported for badgers in hair from this study and from faecal and plasma samples in a study of bears (Cattet *et al.,* 2014), but substantially higher than levels reported from both claw and hair in the American marten (Martes americanus), another mustelid species (Keogh *et al.,* 2022). These lower claw values may result from only the “free” cortisol fraction (i.e. the fraction that is not bound to circulating cortisol-binding globulin and albumin) being able to diffuse into growing claw tissue. The mechanism by which cortisol accumulates in claw and hair structures is not well understood, nor are the effects of recent stress on cortisol measurements in these tissues. In brown bears (Ursus arctos), hair cortisol concentrations were 2.5 times higher in bears sampled by active remote drug delivery (darting) compared to those sampled passively with barbed wire traps; while in polar bears (Ursus maritimus) blood and fat contamination of hair samples has been reported (Bechshøft *et al.,* 2011). Pharmacological challenges with adrenocorticotropic hormone (or ACTH: the pituitary regulator of GC secretion) in captive caribou and reindeer (Rangifer tarandus) failed to affect hair cortisol (Ashley *et al.,* 2011). However, repeated ACTH treatment in Canadian lynx (Lynx canadensis) did increase hair cortisol (Terwissen *et al.,* 2013). The lower levels of cortisol in badger claw versus hair samples may thus reflect the latter being more susceptible to recent stress. Hair samples are potentially swamped with systemic cortisol during trapping, as part of a multipronged stress effect on badgers (Schutz *et al.,* 2006). This could also explain why despite hair and claw samples being correlated, there was more variability in hair concentrations compared to claw. Another consideration is that compared to hair, mammalian claws are structurally reinforced by other substances embedded in the keratin matrix to harden them for digging, and these may serve to “dilute” the cortisol present thus decreasing its measurable concentration. Furthermore, claw samples represent more recent growth whereas hair represents cortisol during the molt period which in badgers occurs from July to December (Maurel *et al.,* 1986) and this may be reflected in differences in concentrations being measured. However no differentiation in the correlation of hair and claw cortisol by season is apparent. Future studies should aim to clarify the relationship between hair and claw hormones in a systematic way.

Badger claw colour ranges from black to a pale straw yellow, due to varying degrees of melanin pigment deposition (Ethier *et al.,* 2010), however when compared no differences in cortisol were observed between claws with varying levels of pigmentation. Many mammalian studies have reported that hair colour can influence recorded cortisol levels, with typically darker fur having higher cortisol levels than light fur (Charalambous and Narayan, 2019; Bowland *et al.,* 2020; Otten *et al.,* 2021; Shi *et al.,* 2021; Braun *et al.,* 2022), possibly due to weak binding to melanin (Binz *et al.,* 2018; Otten *et al.,* 2021). To our knowledge this is the first study reporting variation in claw cortisol relative to colouration, and despite the non-significant results here, future studies in other species are needed.

### Claw cortisol varies between sexes and with season

Male badgers had higher claw cortisol concentrations than did females in both analyses. Sex variation in cortisol has been reported in other mammals, but these are typically sexually dimorphic species with a polygynous mating system, whereas badgers exhibit little sexual size dimorphism (Abramov and Puzachenko, 2005; Butora *et al.,* 2018; Sugianto *et al.,* 2019b). In high-density populations, including Wytham Woods, badgers have a highly promiscuous and polygynandrous mating system (Dugdale *et al.,* 2007) with high rates of extra-group paternity (Annavi *et al.,* 2014a). Although males do not contest mating opportunities, they do mate frequently (Dugdale *et al.,* 2011), where superfecundation and superfetation (Yamaguchi *et al.,* 2006) during delayed implantation (Sugianto *et al.,* 2021) causes a prolonged mating season with prime-age males attaining high testosterone titers with descended testes from the January mating peak, through to October (Buesching *et al.,* 2009). This was reflected in the coarse analysis, where the sex effect was driven largely by high claw cortisol concentrations among males in spring, with the concentrations being more similar between the sexes through the rest of the year. Similarly, inclusion of season and its interaction with sex in the second most supported model in the fine-scale analysis, corroborated convincingly that cortisol concentrations differed significantly between the sexes only during spring. Badger populations in Ireland have similarly reported highest cortisol levels during the spring–summer, for both serum and faecal samples although no sex differences were reported in that study (Agnew *et al.,* 2016). Scent marking also increases during the mating season when badger subcaudal glands produce more secretions with different chemical compositions from the rest of the year (Buesching *et al.,* 2002a, 2002b). Thus high-frequency mating and associated scent marking behaviours may constitute a stressor for males, inducing cortisol production, and associated with a loss of body-condition in the summer months (Woodroffe and Macdonald, 2009; Bright Ross *et al.,* 2021). Female–female competition occurs in badgers and can cause reproductive suppression (Woodroffe and MacDonald, 1995). All mature female badgers mate but genetic pedigrees has revealed that, in this Wytham population, individual breeding success decreases with the number of females in the sett; from 72% when 1–3 females are present to 21% with 11–17 females present (Annavi *et al.,* 2014a). This matriarchal competition stressor has been alleviated in this high-density population by socio-spatial redistribution (Macdonald *et al.,* 2004) with breeding females spread out extensively between subsidiary setts and outlying burrows, potentially reducing female cortisol levels through spring and summer.

In autumn, following mating and conception, females had higher claw cortisol levels than males, suggesting cortisol may be playing a regulatory role in female reproductive physiology (Whirledge and Cidlowski, 2017). In bovines, cortisol has a positive effect on corpus luteum signalling during implantation and establishment (Komiyama *et al.,* 2008; Duong *et al.,* 2012). Although the effects of cortisol on blastocysts suspended in utero during delayed implantation have not been studied in badgers, in American martens there is a negative relationship between fur cortisol and the presence of blastocysts (Keogh *et al.,* 2022). It is plausible that higher cortisol levels could impact female badger fertility (see Andersen, 2002) when blastocysts implant in late fall (Woodroffe, 1995). Nevertheless, more extreme stress-induced cortisol levels broadly result in profound reproductive dysfunction (Romero *et al.,* 1998; Whirledge and Cidlowski, 2013, 2017). In female badgers stress experienced by mothers, even while the implantation of their blastocysts is suspended, can affect later-life telomere length in offspring (van Lieshout *et al.,* 2021b) and susceptibility of offspring to later-life mustelid herpes virus (MusGHV-1) reactivation (Tsai *et al.*, 2021). Higher autumnal cortisol levels may also be linked to hibernation physiology in badgers (Newman *et al.,* 2011), where high cortisol levels reduce activation of the AMPK/PGC-1α/PPAR-α axis in the regulation of metabolism in skeletal muscle and adipose tissue (Vella *et al.,* 2020).

It is important to note, however, that cortisol has many pleiotropic effects including non-stress related functions, such as mobilizing energy reserves for general metabolism, and thus we should be cautious in interpreting this sex difference from a purely stress-context (MacDougall-Shackleton *et al.,* 2019).

### Claw cortisol increases with social group size in males

The inclusion of social group size improved both the coarse and fine-scale analyses, however only for the latter was group size a significant component predictor of claw cortisol concentrations, but this effect was limited to males. One explanation for a lack of effect in the coarse analysis might be that population density was high across all groups, at around 41 badgers km^2^ at the time of this study (Bright Ross *et al.,* 2020). Permanent dispersal rates are low in the Wytham badger population (19%; Macdonald *et al.,* 2008), yet temporary visits between groups are frequent (Ellwood *et al.,* 2017), spreading social stress homogeneously both within and among groups (Macdonald *et al.,* 2004; Macdonald *et al.,* 2015); and thus limiting the effect of social grouping on claw cortisol levels. An alternative interpretation for a lack of significance of group size in the coarse analysis may be that transient spikes in circulating hormones are short term and unless very frequent, would be difficult to detect in claw cortisol samples that are representative of longer-term levels.

In the fine-scale analysis, larger group size was related to higher claw cortisol concentrations in males, but not females. Male badger development is more sensitive to group size, whereas females are more sensitive to resource availability (weather-moderated food supply and energy expenditure; Sugianto et al., 2019). Among adult badgers there is little evidence for social hierarchies in Wytham Woods (Macdonald *et al.,* 2002). However, dominance structures in this species are context dependent, depending on group dynamics and annual resource availability, and when dominance occurs in badgers, females initiate more and receive less aggression than males (Hewitt *et al.,* 2009). In adult males, paternity success appears opportunistic, linked to contact rates with females both within and outside the group (Annavi *et al.,* 2014b). Claw cortisol was highest for male badgers in large groups, potentially reflecting greater competition for mates inducing chronic stress. Studies in other species that exhibit more robust dominance hierarchies may, however, uncover an alignment of claw cortisol with social structure (Sherman and Mehta, 2020; for example as seen in wild chimpanzees (*Pan troglodytes schweinfurthii*)—Muller and Wrangham, 2004).

### Claw cortisol varies with age and body condition

Younger badgers had lower claw cortisol levels than did older individuals in the fine scale analysis, with a similar non-significant trend in the coarse analysis. Stress can interfere with the sex steroid signalling necessary for pubertal sexual development (Whirledge and Cidlowski, 2013). Badgers become sexually mature between 18 and 22 months of age with endocrine puberty commencing in juveniles around 11 months (Sugianto *et al.,* 2019a), and thus maintaining low cortisol concentrations during this developmental stage is likely advantageous. Interestingly, some of the highest claw cortisol levels were also observed among juveniles, resulting in the widest range of claw cortisol titers among individuals for any age class. This may suggest that some juveniles experience substantially more stress than others, although this was not reflected in deviations in body condition or fat scores from age class-specific ranges. Furthermore, male badger cubs exhibit two endocrine phenotypes: an early and late onset of sexual development and associated growth rate (Sugianto *et al.,* 2019a). Notably, four of the five juveniles with high cortisol concentrations exceeding 30 ng/g were males. (Bilham *et al.,* 2017) report that badger cubs trade-off preventing oxidative tissue damage against growth, particularly as juvenile survival probability decreases with oxidative damage. Whether increased claw cortisol is directly associated with either juvenile survival or delayed growth from cortisol-induced oxidative stress in badgers warrants further study.

Higher claw cortisol in older badgers observed here are comparable to a study of claw cortisol in seals (Crain *et al.,* 2021), but in contrast to a study on badgers in Ireland that found no relationship between badger age and faecal cortisol metabolites (Agnew *et al.,* 2016). Higher chronic stress-induced cortisol among the oldest badgers is plausible as such age-related trends have been observed in other social mammals (reviewed in Whirledge and Cidlowski, 2017). Given that age does not relate to dominance in this badger society another plausible explanation for the elevated cortisol in older badgers is senescence, i.e. reduced performance with ageing (Bright Ross *et al.,* 2020). Approximately 13% of badgers in this population survive to be nine and older, an age at which (Sugianto *et al.,* 2020) found that 54.5% of males and 63.6% of females sampled experience andropause/menopause (i.e. reproductive hormone levels dropped to pre-pubescent levels), but still continued to live 0.42 to 4.75 years after reproduction ceased. Elderly badgers suffer substantial loss of body-condition after hot summers and wet winters and experience higher rates of mortality than younger age classes (Bright Ross *et al.,* 2020). That higher claw cortisol was correlated with lower body condition in sexually mature individuals suggests cortisol may either have a potential role or is impacted by lowered condition that occurs with increasing age in this species, however further research in this area is necessary.

## Conclusions

Claw cortisol concentrations correlated with potential sources of chronic stress in this extensively studied badger population, suggesting that the cortisol concentrations established using this claw-based technique may be valid indicators of biologically relevant stressors. As applied specifically to the conservation of badgers, better understanding their chronic stress can inform questions on their management, and particularly the immunosuppressive effects of cortisol may possibly be linked to their role as a reservoir for bovine tuberculosis (bTB). As a counterforce, the sanctioned culling of badgers aimed at controlling bTB incidence is unpopular with conservationists (Lodge and Matus, 2014), leading to calls to better understand and mitigate the stresses involved in badger management (Woodroffe *et al.,* 2005; McCulloch and Reiss, 2017; Jones *et al.,* 2018; Langton *et al.,* 2022).

More broadly, many animal species are threatened by aspects of Human Induced Rapid Environmental Change (Sih, 2013), including direct attempts to manage populations (Swaisgood, 2007), especially when human-wildlife conflicts arise. We advocate the broad utility of this claw cortisol technique as a tool to measure not only the stressful impacts animals experience, but also to accurately quantify the long-term effectiveness of remedial conservation and welfare interventions.

## Funding

This work was supported by an Ashforth Research Grant through Rollins College to HBF and by funding to the WildCRU in support of the Badger Project. CN was funded by the H.N. Southern Fellowship in Ecology.

## Data Availability

The data underlying this article will be shared on reasonable request to the corresponding author.

## Author Contributions

H.B.F conceived the design of this study, with extensive feedback from C.N. and C.D.B. Logistical support was provided by D.W.M. Field data collection was conducted by C.N., C.D.B. and H.B.F. Laboratory analysis was performed by T.B. under the supervision of H.B.F. H.B.F. conducted statistical analyses and wrote the first draft of the manuscript with extensive edits from all other authors.

## Conflict of Interest

The authors have no conflicts to declare.

## Supplementary Material

Web_Material_coad024
