# Supplementary material for: Assessing chronic stress in wild mammals using claw-derived cortisol: a validation using European badgers (Meles meles)
Source: Conserv Physiol. 2023 May 10;11(1):coad024. doi: 10.1093/conphys/coad024 (PMC10171820; doi:10.1093/conphys/coad024)
Supplement: Web_Material_coad024 [file web_material_coad024.zip › Fokidis. BadgerStudy R2 Supplementary Data.pdf]

Table S1. Description and ranges for attributes and variables for describing claw cortisol concentrations in European badgers using both the generalized linear mixed models and mixed models for repeated measures. * Indicates that log transformations were necessary prior to analysis.

Attributes	Variable	Description
Demographics	ID	*Factor:* Unique tattoo number (*fine-scale analysis only*)
	Sex	*Factor:* Male and Female
	Age*	*Covariate:* 3 to 187 months *
Body Condition	BCI	*Covariate*: Regression residuals from -4.484 to 3.980
	Fat	*Covariate*: Score from 1 (emaciated) to 5 (obese)
Reproductive Status	Genitalia	*Factor*: Males - very ascended, ascended, intermediate, descended, or very descended testes, Females - dry or moist vulva
	Lactation	Factor: Females only - not lactated, lactated, and lactating
Socio-spatial	Sett	*Factor*: 61 separate sett locations in study area
	GroupSize*	*Covariate*: 2 to 27 individuals based on minimal number alive estimate for social groups *
Temporal	Season	*Factor*: Spring, Summer, and Autumn
	Year	*Covariate*: 2017 to 2019
Captures	Trap*	*Covariate*: 1st to 10th capture
